# Behavioural intervention for weight loss maintenance versus standard weight advice in adults with obesity: A randomised controlled trial in the UK (NULevel Trial)

**DOI:** 10.1371/journal.pmed.1002793

**Published:** 2019-05-07

**Authors:** Falko F. Sniehotta, Elizabeth H. Evans, Kirby Sainsbury, Ashley Adamson, Alan Batterham, Frauke Becker, Heather Brown, Stephan U. Dombrowski, Dan Jackson, Denise Howell, Karim Ladha, Elaine McColl, Patrick Olivier, Alexander J. Rothman, Alison Steel, Luke Vale, Rute Vieira, Martin White, Peter Wright, Vera Araújo-Soares

**Affiliations:** 1 Institute of Health & Society, Newcastle University, Newcastle upon Tyne, United Kingdom; 2 Fuse, the UK CRC Centre for Translational Research in Public Health, Institute of Health & Society, Newcastle University, Newcastle upon Tyne, United Kingdom; 3 Human Nutrition Research Centre, Newcastle University, Newcastle upon Tyne, United Kingdom; 4 School of Psychology, Newcastle University, Newcastle upon Tyne, United Kingdom; 5 Centre for Rehabilitation, Exercise and Sport Sciences (CRESS), Teesside University, Middlesbrough, United Kingdom; 6 Health Economics Research Centre, University of Oxford, Oxford, United Kingdom; 7 Faculty of Kinesiology, University of New Brunswick, Fredericton, Canada; 8 Open Lab, School of Computing Science, Newcastle University, Newcastle upon Tyne, United Kingdom; 9 Department of Psychology, University of Minnesota, Minneapolis, Minnesota, United States of America; 10 Newcastle Clinical Trials Unit, Newcastle University, Newcastle upon Tyne, United Kingdom; 11 Centre for Diet and Activity Research (CEDAR), MRC Epidemiology Unit, School of Clinical Medicine, University of Cambridge, Institute of Metabolic Science, Cambridge, United Kingdom; Chinese University of Hong Kong, CHINA

## Abstract

**Background:**

Scalable weight loss maintenance (WLM) interventions for adults with obesity are lacking but vital for the health and economic benefits of weight loss to be fully realised. We examined the effectiveness and cost-effectiveness of a low-intensity technology-mediated behavioural intervention to support WLM in adults with obesity after clinically significant weight loss (≥5%) compared to standard lifestyle advice.

**Methods and findings:**

The NULevel trial was an open-label randomised controlled superiority trial in 288 adults recruited April 2014 to May 2015 with weight loss of ≥5% within the previous 12 months, from a pre-weight loss BMI of ≥30 kg/m2. Participants were self-selected, and the majority self-certified previous weight loss. We used a web-based randomisation system to assign participants to either standard lifestyle advice via newsletter (control arm) or a technology-mediated low-intensity behavioural WLM programme (intervention arm). The intervention comprised a single face-to-face goal-setting meeting, self-monitoring, and remote feedback on weight, diet, and physical activity via links embedded in short message service (SMS). All participants were provided with wirelessly connected weighing scales, but only participants in the intervention arm were instructed to weigh themselves daily and told that they would receive feedback on their weight. After 12 months, we measured the primary outcome, weight (kilograms), as well as frequency of self-weighing, objective physical activity (via accelerometry), psychological variables, and cost-effectiveness. The study was powered to detect a between-group weight difference of ±2.5 kg at follow-up. Overall, 264 participants (92%) completed the trial. Mean weight gain from baseline to 12 months was 1.8 kg (95% CI 0.5–3.1) in the intervention group (*n* = 131) and 1.8 kg (95% CI 0.6–3.0) in the control group (*n* = 133). There was no evidence of an effect on weight at 12 months (difference in adjusted mean weight change from baseline: −0.07 [95% CI 1.7 to −1.9], *p* = 0.9). Intervention participants weighed themselves more frequently than control participants and were more physically active. Intervention participants reported greater satisfaction with weight outcomes, more planning for dietary and physical activity goals and for managing lapses, and greater confidence for healthy eating, weight loss, and WLM. Potential limitations, such as the use of connected weighing study in both trial arms, the absence of a measurement of energy intake, and the recruitment from one region of the United Kingdom, are discussed.

**Conclusions:**

There was no difference in the WLM of participants who received the NULevel intervention compared to participants who received standard lifestyle advice via newsletter. The intervention affected some, but not all, process-related secondary outcomes of the trial.

**Trial registration:**

This trial is registered with the ISRCTN registry (ISRCTN 14657176; registration date 20 March 2014).

## Abstract 2

Falko Sniehotta and colleagues reveal their trial results where face-to-face goal setting can be an effective intervention for weight loss, but in the longer term these measures aren't effective in keeping weight off.

## Author summary

**Why was this study done?:**

- Obesity is a major contributor to preventable life-years lost worldwide.
- Effective behavioural weight loss interventions are widely available, but weight loss is often followed by weight regain.
- Effective interventions to slow down weight regain are intensive and not widely available. There is a need for accessible, effective interventions that can support individuals who have lost weight.

**What did the researchers do and find?:**

- We carried out a randomised controlled trial (RCT) involving 288 people with obesity who had recently lost weight. Participants were given wirelessly connected weighing scales, and they were randomly assigned into 2 groups and studied over 12 months.
- One group received standard lifestyle advice via newsletter and no feedback on their use of the scales. The other group received the NULevel intervention, consisting of a single face-to-face meeting and subsequent weight maintenance support delivered via short message service (SMS) messages with embedded links. The intervention was developed based on maintenance theory and effective behavioural principles such as self-monitoring, goal setting, feedback, and reinforcement.
- Intervention participants weighed themselves more frequently than control participants and were more physically active, but we found no difference in weight regain between the 2 groups at 12 months.

**What do these findings mean?:**

- The study shows that the NULevel intervention on its own does not seem to slow the rate of weight regain.
- Further work is required to identify the optimal set and dose of intervention strategies needed to effectively support people who have lost weight in keeping it off.

## Introduction

Helping people with obesity to avoid weight regain after clinically significant weight loss (≥5%) [1] is vital for tackling the increasing global burden of obesity-linked preventable morbidity and mortality [2]. Effective behavioural weight loss interventions are widely available [3], but interventions to support individuals in maintaining weight loss that are scalable for population delivery and impact are not [4]. Maintenance interventions are needed because obesity is a chronic, relapsing condition in which a third of weight loss is typically regained in a year and the rest within 3 to 5 years [5,6]. This rate of recidivism greatly attenuates the health and economic benefits of weight loss [7] and has been branded the most substantial current problem in obesity management [8].

Trials of evidence-based interventions to support adults with obesity in weight loss maintenance (WLM) are rare, heterogeneous, and of variable quality [4]. A recent meta-analysis of such trials concluded that intensive lifestyle interventions, targeting both dietary and physical activity behaviours, can effectively slow down weight regain in these individuals [4]. Most trials began by inducing weight loss in participants, before offering maintenance support to those who lost a specified amount of weight [9,10], limiting their generalisability to only individuals who responded well to a particular weight loss treatment. As such, much of the existing evidence base for WLM does not take into account the wide variety of methods by which individuals with obesity initially lose weight. Few previous studies have recruited participants who undertook initial weight loss independently of the maintenance intervention programme [11–13]. Moreover, although effective, these interventions involved multiple one-to-one or group-based participant contacts over prolonged periods, which may reduce their cost-effectiveness and limits their scalability. Systematic review evidence does not suggest that internet interventions are more effective than control conditions in reducing weight regain [4].

Mobile internet technology can potentially provide individually tailored behavioural weight management support at scale [14,15] and link with wirelessly connected personal weighing scales for weight self-monitoring [16], but this combination has not been used in previous WLM trials. Regular self-weighing appears to be a beneficial component of WLM interventions [11,17], as does the use of dietary and physical activity behavioural strategies based on self-regulation theory [11,12,18]. A systematic review of existing evidence [4] found insufficient evidence to conclude whether more intensive versions of lifestyle interventions are more effective than less intensive versions [13,19]. Lower intensity interventions delivered via mobile internet technology, incorporating regular self-weighing and self-regulatory behavioural strategies, may address the need for flexible, scalable WLM interventions for adults with obesity who have achieved clinically significant weight loss.

Our aim was to determine whether a lower-intensity, mobile internet technology-assisted behavioural intervention could reduce weight regain among adults with obesity with clinically significant weight loss achieved outside of a research context and whether such an intervention is cost-effective, compared to standard lifestyle advice.

## Methods

A full protocol detailing the trial methods has been published previously [16]. We obtained ethics approval from the East Midlands-Derby National Research Ethics Service (REC: 14/EM/0069; 6 February 2014). Individual participants provided written informed consent prior to commencement of the baseline measurements. The trial was registered on the ISRCTN registry on 20 March 2014 (ISRCTN14657176: http://www.controlled-trials.com/ISRCTN14657176). Any adverse events were monitored and recorded by the trial administrator using standard procedures of the Clinical Trials Unit.

### Participant screening and recruitment

A total of 288 participants were recruited from a range of sources across North East England between 28 April 2014 and 27 May 2015. Major sources of recruitment comprised commercial weight loss providers (17.3%), word of mouth (15.6%), social media (15.3%), and local employer staff websites (15.1%). Other sources included the university’s public-facing homepage (6.9%), local council public websites and newsletters (6.6%), invitation letters sent to participants in previous (unrelated) university research (5.6%), local flyers and posters (2.1%), local radio and newspaper advertisements (2.1%), and a local authority-commissioned weight management programme (1.0%). A further 1.4% of participants were recruited through other means, and 10.1% did not specify the recruitment channel.

Individuals were eligible to take part if they were aged ≥18 years, had a body mass index (BMI) of ≥30 kg/m2 in the 24 months preceding trial entry (≥28 kg/m2 for individuals of South Asian descent), and had lost ≥5% body weight in the 12 months preceding trial entry. Individuals were requested to provide written verification of weight loss from a physician, weight loss counsellor, or friend or family member; if this was unavailable, then participants self-certified their weight loss. To participate, individuals needed to be able to use a standing scale, to be willing and able to attend study visits at Newcastle University, and to have use of an internet-enabled mobile telephone.

Individuals were ineligible to take part if they had lost weight through illness or surgical procedures or were pregnant, planning to become pregnant during the study period, or breastfeeding an infant <6 months old. Other exclusion criteria were current involvement in other weight research studies, an inability to understand written or spoken English, a diagnosis of an eating disorder or condition that significantly limited physical activity, a baseline weight of >175 kg (due to capacity limitations of the study scales), and plans to leave the geographical area for a prolonged time during the study period. A data collector, blinded to subsequent randomised group allocation, enrolled participants in the trial.

### Randomisation and blinding

A researcher used a secure web-based randomisation system to allocate eligible, consenting participants to 1 of 2 groups after they completed baseline assessment, to receive either standard lifestyle advice via newsletter (control group) or the behavioural intervention (intervention group). Randomisation occurred in a 1:1 ratio (144 in each arm) and was stratified by sex and prior weight loss (<10% versus ≥10%). Concealment of allocation was achieved using the web-based randomisation system, based on variable-length blocks, provided by the Newcastle Clinical Trials Unit via Data Architects Ltd. (Newcastle upon Tyne, UK). Research staff involved in assessing the study outcomes were blinded to the allocation of participants. Participants were asked not to reveal their trial allocation at follow-up assessments, and instances in which participants divulged their allocation status (3 participants in the intervention and 1 in the control arm) were documented. The statistician undertaking data analysis was unblinded.

### Allocation of study scales

All participants received a set of digital-display wireless body-weight scales at the baseline appointment and were shown how to use them. Every time participants weighed themselves, the scales sent the recorded weight over the mobile phone network to the online study interface via an internal multinetwork SIM card. Weights were automatically recorded, dated, and time-stamped for each participant. Participants were informed that all weight data were recorded, but only those allocated to the intervention arm would receive feedback on their weight progress.

### Intervention

The intervention development process followed Medical Research Council guidance for the development of complex interventions [20] and has been described previously [16]. Briefly, intervention content was designed in line with self-regulation theory [21] within the context of the health action process approach [22,23]. Intervention content also drew on the features of previously effective self-regulation WLM interventions [11], a systematic review of theories of behavioural maintenance [24], and findings of a systematic review and meta-analysis of WLM interventions [4]. We used phone-based mobile internet technology to help participants monitor their weight, set behavioural goals, track goal progress, and plan for risk factors for regain and to provide feedback and reinforcement, drawing on effective behavioural principles [25]. The intervention was delivered using the combination of a single face-to-face meeting with an intervention team member and regular automated short message service (SMS) (at least 1 every 2 days) with embedded links and other content (triggered by participants’ weight and weekly online-questionnaire data), along with personalised SMS generated by the intervention team. Individual telephone calls with a member of the research team could be scheduled on participant request. The core intervention components are summarised in Box 1.

**Box 1. NULevel Intervention components**

**Daily weighing with wirelessly connected scales:** All participants received a set of digital body-weight scales at the baseline assessment appointment. The scales featured an embedded SIM card, which wirelessly transmitted each weight over the mobile phone network to the online study interface. Intervention participants were asked to weigh themselves daily and received feedback on their weight progress by SMS; control participants received no instructions or feedback.

**One-to-one consultation:** Soon after randomisation, intervention participants attended a single one-to-one consultation at Newcastle University with a trained facilitator (psychologist) to help support the transition from weight loss to WLM. The facilitator evaluated the sustainability of participants’ current diet and physical activity for WLM and supported them in setting maintenance goals and developing action plans for weight, diet, and physical activity. Participants were taught to self-monitor their weight, diet, and physical activity goal progress using the online study interface and a pedometer (provided). Participants also received support in planning for scenarios with a high risk of behavioural lapses and concomitant weight regain.

**Online study interface:** Participants were prompted to log on to an online study interface regularly, to view a real-time weight graph and record weekly progress towards dietary and physical activity goals (self-monitoring). They could also request further contact with the intervention team. The interface facilitated the provision of intervention content via SMS (see below).

**SMS text messages and support:** Participants received tailored, automated SMS feedback on their recent weight, dietary, and physical activity goal progress from the online study interface. Participants with a stable weight received only minimal reinforcement, whereas participants with regain received more intensive support and encouragement to re-engage with weight control strategies. SMS reminders were sent to prompt participants to weigh themselves and complete their weekly diary. The participant and research team used SMS for ad hoc, unscripted communications. Finally, participants also received an automated schedule of SMS to support WLM over the course of the intervention, with links and other embedded content drawing on theoretical themes of behavioural maintenance.

Intervention participants were encouraged to weigh themselves daily and use the online study portal to monitor their weight on a graph showing the weight data sent by their scales. When the intervention software detected weight changes, the online study interface sent participants automated feedback via SMS. In this way, feedback was tailored to participants’ weight trajectory, providing low-intensity positive reinforcement when the body weight was stable and higher intensity prompts to re-engage with weight control strategies when body weight increased above a threshold specified by the individual participant. Participants met a research team member (psychologist) once, for around an hour, to learn about the intervention and receive support to set and plan for behavioural goals (diet and physical activity), plan for relapse prevention, and to learn how to self-monitor their diet, physical activity, and weight in the transition from weight loss to WLM. The development of this consultation has been described elsewhere [26]. Participants were given a pedometer (Omron UK Ltd, Milton Keynes, UK) and prompted to record their progress towards physical activity goals (step counts) and dietary goals in a weekly diary on the study interface. When data were entered, automated feedback on behavioural goal progress was sent by the online study interface via SMS.

### Control

Participants in the control arm did not receive any instructions regarding frequency of use for the study scales although they were made aware, for ethical reasons, that the study team could see their weight data. They received standard lifestyle advice on 4 occasions, 3 months apart, delivered in SMS with embedded links. Content was drawn from the NHS Choices website (www.nhs.uk/livewell) and included information on healthy food swaps, 100-calorie snacks, healthy breakfasts, and how to read nutritional labels. Intervention participants also received these 4 messages as part of their automated schedule of SMS. Other than to arrange follow-up assessment, no further scheduled contact with the control group occurred.

### Outcome assessment

All outcome assessments were made by research staff not involved in any other aspect of the study and blinded to the participants’ group allocation. Outcome assessment took place at Newcastle University or at a community hall venue. Participants received £25 shopping vouchers for attendance at baseline and £25 shopping vouchers for attendance at 12-month follow-up. Height, age, and gender were measured at baseline; all other measurements were taken at baseline and 12-month follow-up.

### Measures

The primary outcome was change in weight (kilograms) from baseline (i.e., randomisation) to 12 months. Body weight, clothed without shoes, was measured to the nearest 0.1 kg using digital portable scales (SECA model 875; SECA UK Ltd, Birmingham, UK). Height was measured to the nearest 1 mm using a Leicester Height Measure stadiometer (SECA UK Ltd). BMI was calculated as body weight (kilograms) divided by the square of height (metres). Waist circumference and hip circumference were recorded with an anthropometric tape measure, following established protocols [27]. Body fat percentage was measured using an Omron BF306 handheld body fat monitor (Omron UK Ltd, Milton Keynes, UK), and resting heart rate and blood pressure were measured using an Omron HEM-7200 arm cuff automatic blood pressure monitor (Omron UK Ltd). Physical activity was assessed using an ActiGraph GT3X+ (ActiGraph, LLC., Pensacola, FL) accelerometer worn for at least 8 hours per day over at least 4 days. The outcome variable was total activity counts per day (TAC/d) irrespective of intensity, and data were recorded at epochs of 1 minute [28].

Participants completed questionnaires to measure health-related quality of life, assessed using the EQ-5D-3L [29], and healthcare costs and service usage, assessed using a structured questionnaire that was designed bespoke for this study from an existing item bank and a database of tools (www.dirum.ac.uk). These data were used to estimate quality adjusted life years (QALYs) and costs, respectively, and then the incremental cost per QALY gained. Satisfaction with weight outcomes was assessed using the Weight Outcomes Satisfaction Scale [30]. Self-efficacy for healthy eating, physical activity, and WLM was measured using adaptations of existing questionnaires [31,32], as were action planning and coping planning [33]. Automaticity of healthy eating, physical activity, and self-weighing were assessed using adapted versions of the Self-Report Behavioural Automaticity Index [34,35]. Use of self-regulation strategies for WLM was measured via the Regulatory Focus Questionnaire [23,36,37], ego depletion was assessed using a questionnaire developed for this study, and social support was measured using the Enhancing Recovery in Coronary Heart Disease (ENRICHD) Social Support Inventory [38]. We also compared frequency of self-weighing in both trial arms (as automatically recorded, date-stamped, and time-stamped on the study interface).

### Fidelity assurance

The fidelity of delivery of the face-to-face meetings was assessed by coding 20 (15% of 131 sessions) randomly selected audio recordings. A coding scheme was developed from the intervention session manual and included 15 prespecified intervention components (e.g., introduction, agenda setting, review of recent weight loss) and 15 prespecified behaviour change techniques (BCTs) from the ‘Coventry, Aberdeen & London-Refined’ (CALO-RE) taxonomy (e.g., goal setting for outcome and behaviour, action planning, barrier identification). The coding scheme was developed, and all coding completed by one author (SUD) who had not been involved in the delivery of the intervention. Fidelity scores were summarised per component and BCT (across sessions) and per session (across components/BCTs), and an average fidelity score across sessions and components/BCTs was computed.

### Statistical analysis

Sample size calculations estimated that 2 groups of 122 participants providing data on the primary outcome (weight change at 12 months post randomisation) were required to detect a 2.5 kg between-group mean difference with 90% statistical power, given a type 1 error rate of 5% and assuming a standard deviation of weight change of 6 kg. Assuming a rate of 15% loss to follow-up, a total sample of 288 randomised participants was needed. The parameters of this power calculation were derived from a systematic review of comparative behavioural WLM trials [4].

All analyses were conducted according to the intention-to-treat principle, using STATA (version 14.1) statistical software. We used univariate descriptive statistics to summarise the characteristics of the study sample at baseline. We used multivariate linear regression analyses to compare the intervention and control groups on weight at 12 months post randomisation, adjusting for baseline weight, stratification variables (sex of the participant and a binary indicator of whether the participant lost more than 10% of their body weight), and index of multiple deprivation (IMD) [39]. Results were in the form of a 95% confidence interval for the mean difference in weight between participants randomised to the intervention arm and participants randomised to the control arm. Where the standardised residuals were not normally distributed, an alternative confidence interval was calculated using resampling (bootstrap) procedures. Secondary outcomes were analysed using the same approach, with an appropriate error structure adopted for each particular measure.

### Health economic analyses

A within-trial cost-utility analysis was used to compare the NULevel intervention with usual practice. Trial data and seemingly unrelated regressions (SURs) were used to estimate costs (associated with the delivery of the intervention, healthcare, private healthcare, use of gyms, and fitness classes) in 2015 pounds sterling (£), and outcomes of the intervention in terms of changes in health-related quality of life were measured by the EQ-5D-3L and compared to usual practice over a 12-month follow-up. From these data, the incremental cost per QALY gained at 12 months was calculated. Sensitivity analyses accounted for a potential effect of reductions in salary costs associated with the delivery of the intervention.

## Results

### Participant characteristics

**Fig 1:**
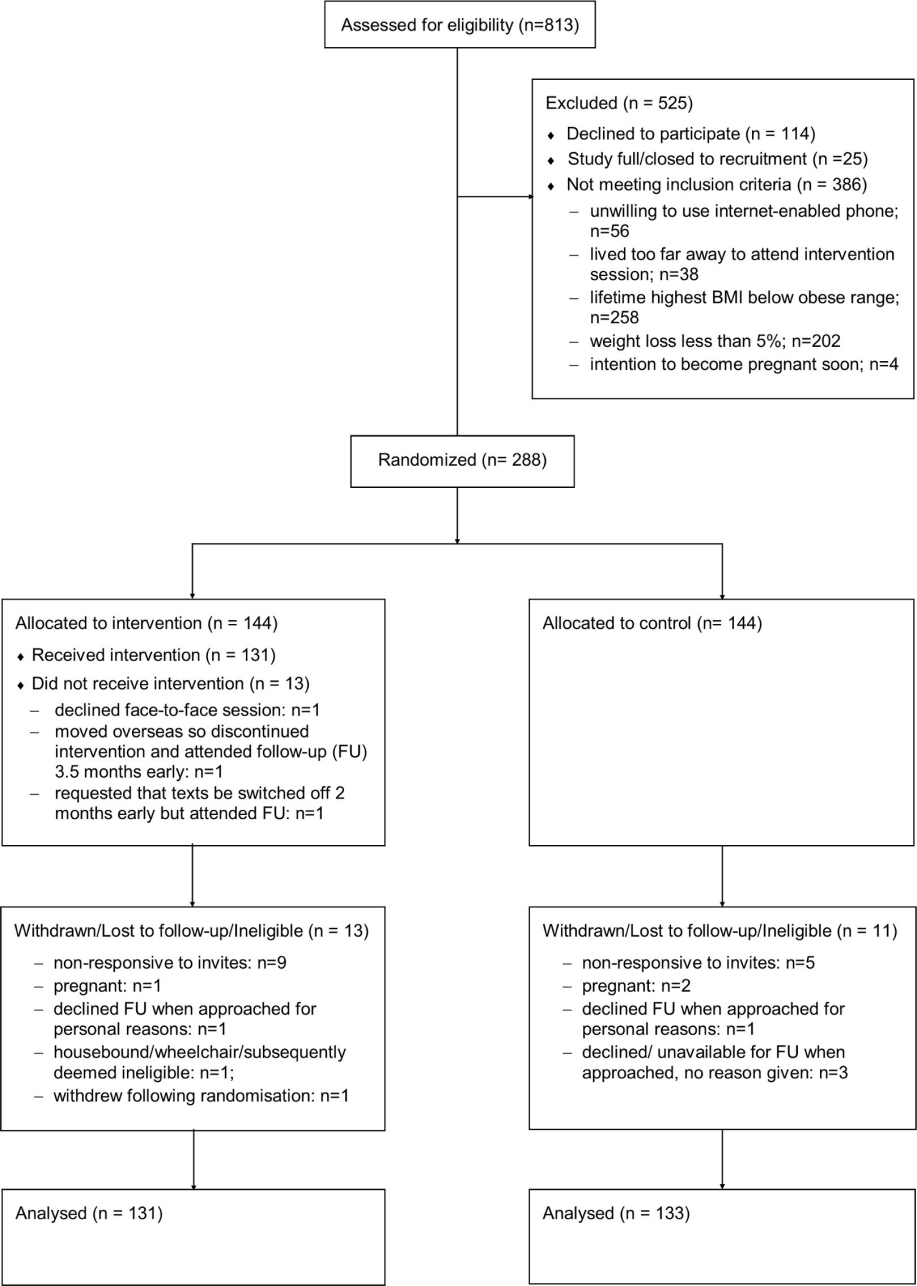
CONSORT 2010 flow diagram.

Between 28 April 2014 and 27 May 2015, 813 individuals volunteered to participate in the NULevel trial and were assessed for eligibility. Reasons for exclusion included ineligibility (386; 47%), non-response, or withdrawal following initial application (n = 114; 14%) and application to join the trial after it had closed to recruitment (n = 25; 3%). Specific reasons for ineligibility are provided in Fig 1, and 122 people were ineligible for 2 or more of the specified reasons. Overall, 288 individuals (34%) were successfully randomised, and Fig 1 shows the flow of these participants through the trial. Forty-eight trial participants (16.7%) provided objective verification of their weight loss prior to trial entry, and 240 self-certified it (83.3%).

The final data collection date for the primary and secondary outcome measures was 24 May 2016. A total of 264 participants took part in the 12-month follow-up, giving a retention rate of 92%; reasons for loss to follow-up are shown in Fig 1. Retention did not differ between the intervention and control groups. Participant baseline characteristics are shown in Table 1 for both study groups. There were no systematic differences between groups at baseline. Physical activity data was available for 226 participants in the 12-month follow-up with a median number of 7 (range: 4 to 11) valid days wearing the accelerometer (control: 6 [4 to 11] and intervention: 7 [4 to 11]). The mean average number (SD) of vector magnitude counts per day at 12 months was 430,503.8 (156,480.4) for the control group and 448,920.3 (140,190.9) for the intervention group.

**Table 1:** Baseline characteristics of all individuals included in the NULevel trial.

Baseline characteristic	Control group (*n* = 144)	Intervention group (*n* = 144)
**Age (y)**	41.6 (11.4)	42.0 (11.6)
**Female, *n* (%)**	113 (78.5%)	110 (76.4%)
**Weight (kg)**	85.5 (15.9)	85.6 (17.5)
**Height (m)**	1.67 (0.09)	1.67 (0.09)
**BMI (kg/m**^**2**^**)**	30.8 (5.2)	30.9 (5.5)
**Pretrial 12-month highest weight (kg)**	99.8 (20.7)	98.2 (19.5)
**Pretrial weight loss (kg)**	−14.4 (11.6)	−12.6 (7.2)
**Pretrial weight loss (% highest)**	−13.9 (7.8)	−12.8 (6.5)
**Waist circumference (cm)**	94.6 (14.7)	93.6 (13.4)
**Hip circumference (cm)**	110.3 (11.6)	110.0 (11.2)
**Systolic BP (mmHg)**	121.4 ± 18.7	123.9 (16.3)
**Diastolic BP (mmHg)**	77.6 (10.1)	76.5 (10.1)
**Resting heart rate (BPM)**	70.6 (11.6)	71.9 (11.0)
**Physical activity (TAC/d)**	476,753 (162,930)	476,615 (160,932)
**Highest level of education, *n* (%)**		
Bachelor’s degree or higher	71 (50.0%)	81 (56.2%)
Post-16 qualification (e.g., HND/A-level)	31 (21.5%)	34 (23.6%)
GCSE/O-level or below	41 (28.5%)	29 (24.3%)
**Employment status, *n* (%)**		
Full employment	88 (61.1%)	87 (60.4%)
Part-time employment	25 (17.4%)	25 (17.4%)
Retired	10 (6.9%)	17 (11.8%)
No paid employment	21 (14.7%)	15 (10.5%)
**Household income, *n* (%)**		
<£10,000	6 (4.2%)	5 (3.5%)
£10,001–£40,000	66 (45.9%)	70 (48.7%)
£40,001–£70,000	52 (36.1%)	50 (34.7%)
>£70,000	20 (13.9%)	19 (13.2%)

Values are mean (SD) for continuous variables and frequencies (%) for categorical variables.

**Abbreviations:** BMI, body mass index; BP, blood pressure; BPM, beats per minute; GCSE/O-Level, General Certificate of Secondary Education/Ordinary Level; HND/A-level, Higher National Diploma/Advanced Level; TAC/d, total activity counts per day.

At baseline, all 288 weight values were obtained by the data collector. At 12-month follow-up, 253 weight values were obtained by the data collector, whilst 11 were obtained from the most recent weight recorded by their allocated SIM-enabled weighing scales (within the previous month). Remote data were used only when the participant had weighed themselves frequently and regularly enough to establish a pattern or had stepped on their scales on the day of completing the questionnaires online, as requested, so we could be confident that the weight was accurate.

No adverse events were reported during the course of this trial.

### Outcomes

Using intention-to-treat model-based regression analyses, we found no evidence of differences in the primary outcome, weight at 12 months from baseline, between the intervention group and the control group, adjusted for baseline weight, stratification variables (sex and prior weight loss) and IMD (see Table 2). Intervention participants mean change was 1.8 kg (SD: 7.4), and control participants’ mean change was 1.8 kg (SD: 7.1). Regression analyses of objective physical activity data, obtained using accelerometers, indicated that both groups became less physically active over the study. There was a small but significant difference between the arms such that intervention group participants were more physically active at 12-month follow-up than control participants, adjusting for baseline levels of physical activity, stratification variables, IMD, and device wear-time. No significant difference in resting heart rate between groups was found at follow-up.

**Table 2:** Weight and physical activity outcomes at baseline and 12 months.

	Control		Intervention		Between-group comparison		
	Baseline	12 mo	Baseline	12 mo	Mean difference (intervention − control)	95% CI	*p-*Value
**Weight (kg)**	85.2 (15.7)	87.0 (16.7)	85.1 (17.5)	86.8 (18.2)	−0.07	1.7 to −1.9*	0.94
**Physical activity (TAC/d)**	476,752.7 (162,929.6)	430,503.8 (156,480.4)	476,614.8 (160,932.4)	448,920.3 (140,190.9)	38,993.2	71,582.4 to 64,03.9	-

Values are mean (SD).

*Bias-corrected and accelerated CIs obtained using bootstrapping due to non-normal distribution of residuals. Note: Between-group comparisons were carried out using multivariable regression analyses, adjusted for baseline, stratification variables, and index of multiple deprivation. Weight comparisons were based on 133 control participants and 131 intervention participants. PA comparisons were based on 86 control participants and 92 intervention participants.

**Abbreviations:** CI, confidence interval; PA, physical activity; TAC/d, total activity counts per day.

Weight data from the wirelessly connected scales used by all participants showed that those in the intervention group weighed themselves more frequently, per week, than control group participants (4.4 [1.4] versus 1.8 [1.7] times per week; mean [SD]). Overall, intervention group participants weighed themselves on an average of 62.8% (20.3) of days spent in the trial; control group participants weighed themselves on an average of 26.1% (24.4) of days.

Table 3 shows mean values and between-group differences for the psychological variables at baseline and 12 months for the 129 control participants and 124 intervention participants who completed the questionnaires at follow-up. Intervention and control groups did not differ on self-efficacy, perceived behavioural control, or automaticity for physical activity, nor did they differ on regulatory focus, ego depletion, or social support. Compared to control participants, intervention participants reported the following: greater satisfaction with weight outcomes; greater habit strength for self-weighing; higher self-efficacy, perceived behavioural control, action planning, coping planning, and automaticity for healthy eating; higher action planning and coping planning for physical activity; and greater confidence for weight loss and WLM.

**Table 3:** Psychological variables at baseline and 12 months.

	Control (*n* = 129)		Intervention (*n* = 124)		Between-group differences	
Variable	Baseline	12 mo	Baseline	12 mo	Mean diff	95% CI
**Satisfaction with weight outcomes (1**–**5)**	4.0 (1.2)	2.7 (1.4)	4.0 (1.1)	3.2 (1.5)	−0.65	−1.0 to −0.3^*^
**Healthy eating self-efficacy (1**–**4)**	2.8 (0.6)	2.6 (0.7)	2.9 (0.6)	2.9 (0.7)	−0.30	−0.48 to −0.15^*^
**Physical activity self-efficacy (1**–**4)**	2.9 (0.8)	2.8 (0.8)	3.0 (0.7)	2.9 (0.7)	−0.09	−0.25 to 0.07
**Healthy eating perceived behavioural control (1**–**7)**	5.1 (1.2)	4.9 (1.4)	5.5 (1.2)	5.5 (1.4)	−0.46	−0.77 to −0.15^*^
**Physical activity perceived behavioural control (1**–**7)**	4.6 (1.5)	4.6 (1.7)	4.7 (1.5)	4.7 (1.8)	−0.17	−0.55 to 0.25^*^
**Weight loss confidence (1**–**7)**	5.2 (1.3)	4.3 (1.6)	5.5 (1.3)	5.0 (1.7)	−0.59	−0.99 to −0.18^*^
**WLM confidence (1–7)**	4.2 (1.6)	4.1 (1.7)	4.3 (1.5)	5.2 (1.7)	−1.10	−1.49 to −0.68^*^
**Action planning (healthy eating; 1–4)**	3.1 (0.9)	2.9 (1.0)	3.3 (0.7)	3.1 (0.9)	−0.24	−0.48 to −0.01^*^
**Coping planning (healthy eating; 1–4)**	2.6 (0.9)	2.5 (1.0)	2.8 (0.9)	2.8 (0.9)	−0.32	**−0.52 to −0.11**
**Action planning (physical activity; 1–4)**	2.7 (1.1)	2.1 (1.0)	2.9 (1.0)	2.4 (1.0)	−0.25	−0.49 to −0.02^*^
**Coping planning (physical activity; 1–4)**	2.2 (0.9)	2.1 (1.0)	2.2 (1.0)	2.4 (1.0)	−0.28	**−0.50 to −0.05**
**Automaticity (healthy eating; 1–4)**	2.6 (0.8)	2.7 (0.9)	2.7 (0.8)	3.0 (0.8)	−0.21	**−0.39 to −0.02**
**Automaticity (physical activity; 1–4)**	2.4 (0.9)	2.4 (1.0)	2.4 (0.9)	2.7 (0.9)	−0.19	−0.39 to 0.01
**Automaticity (self-weighing; 1–4)**	2.7 (1.0)	2.7 (1.0)	2.7 (1.1)	3.1 (1.0)	−0.43	−0.64 to −0.19^*^
**Regulatory focus (promotion; 6–30)**	20.7 (3.3)	20.7 (3.6)	21.4 (3.4)	20.9 (3.3)	0.12	−0.57 to 0.86^*^
**Regulatory focus (prevention; 5–25)**	17.5 (3.8)	17.7 (4.0)	17.2 (3.8)	17.3 (3.6)	0.04	−0.58 to 0.69^*^
**Ego depletion (12–60)**	31.3 (9.0)	34.2 (9.4)	29.9 (8.4)	32.1 (10.0)	1.54	−0.53 to 3.60
**Social support (6–36)**	24.6 (5.4)	24.0 (5.9)	25.5 (4.5)	24.5 (5.8)	−0.31	−1.46 to 0.85^*^

Values are mean (SD). Between-group comparisons were carried out using multiple regression analyses, adjusted for baseline, stratification variables, and index of multiple deprivation. Boldface indicates statistical significance.

*As the residuals were non-normally distributed, this is a bias-corrected and accelerated CI obtained using bootstrapping.

**Abbreviation:** CI, confidence interval; WLM, weight loss maintenance.

### Fidelity of intervention delivery

The fidelity of delivery of both intervention components (97%) and BCTs (92%) was high. Specifically, 12 out of 15 intervention components and 11 out of 15 BCTs were rated as delivered in all sessions. Review of the 4-day food diary was delivered with lower fidelity (75% of sessions), typically because participants did not complete the diary prior to the session or forgot to bring it with them. Agenda setting (90%) and plan for physical activity setbacks (95%) were also not rated as delivered in all sessions, which may reflect a recording issue rather than actual failure to deliver components (i.e., the audio started part-way or stopped recording prior to the conclusion of the session). The BCTs that were not consistently delivered reflected those that were optional and to be delivered only under certain circumstances (e.g., information on where and when to perform the behaviour was used in the context of goal setting if the participant was struggling to generate their own ideas: 55%). It is therefore likely that fidelity was even higher than the ratings of 97% and 92% suggest.

### Economic evaluation

The average total cost (unadjusted) was estimated as £680 (£663) in the intervention group and £583 (£833) in the control group. The SUR estimate of the incremental cost to deliver the intervention was £131 (95% CI: −67 to 338) per participant. The difference in mean QALYs gained between the intervention and control arms after adjusting for baseline EQ-5D-3L was 0.002 (95% CI: −0.014 to 0.018). The probabilities for the intervention to be cost-effective at the standard thresholds of £20,000 to £30,000 for society’s willingness to pay for a QALY gained was between 34% and 41%, implying that it is unlikely that the intervention could be considered cost-effective based on current evidence. This probability increased to around 43% to 47% in a sensitivity analysis accounting for a scenario of 50% lower intervention delivery costs, but the intervention was still likely not to be considered cost-effective in its current form.

## Discussion

In this study, there was no evidence of an effect of a low-intensity behavioural intervention on WLM at 12 months compared to standard lifestyle advice and provision of externally monitored weighing scales in a self-selected sample of women and men with obesity prior to clinically significant weight loss. Participants randomised to receive the intervention had higher levels of objectively measured physical activity at 12 months and weighed themselves more frequently than control participants throughout the study. Intervention participants also differed from controls on multiple psychological variables at 12 months that are thought to be determinants of successful WLM, including greater levels of outcome satisfaction, confidence, and planning.

Our findings of no difference in regain between groups may appear at odds with a meta-analysis [4] that found that lifestyle interventions were associated with lower regain amongst individuals with obesity following weight loss. However, the trials included in the aforementioned analysis differed from the current trial with regard to delivery modality, intervention intensity, and sample composition. Previous successful interventions have tended to involve frequent face-to-face [13] or lengthy telephone-based [10] intervener contacts over prolonged periods of time, greatly limiting scalability, and integration of technological innovations with such approaches to deliver effective interventions at scale is highly desirable [15]. However, similar null results to those obtained in the current trial have been reported in previous lifestyle intervention trials in which internet technology was used to deliver some or all of the intervention content [9,11,40,41]. A recent UK trial of the SMS-supported weight maintenance programme ‘Lighten Up Plus’ found no evidence of effectiveness of the programme at 3 or 9 months [42]. These findings indicate that there is a balance to be struck between scalability, effectiveness, and cost-effectiveness and that the highest value based on the current evidence may be obtained from interventions involving repeated personal contact.

The absolute amount of weight regained by the NULevel intervention group resembles the regain of the intensive intervention group of a study by Wing and colleagues [11], which utilised a similar population and theoretical perspective as the current trial. In contrast, the 12-month regain of the NULevel no-intervention control group was significantly lower than expected when compared to control arms in similar randomised controlled trials (RCTs) [11,12]. This may be partially explained by the relatively high level of self-weighing (twice weekly, on average) amongst the control group, as objectively recorded by the wirelessly connected study scales. The provision of wireless body weight scales to both, intervention and control arm in NULevel with the information that the study team could see participants’ weights might have acted on its own as an active intervention in the control arm. Previous WLM RCTs almost exclusively relied on no-intervention control arms, and none recorded objective self-weighing in both groups [4]. Madigan and colleagues reviewed self-weighing interventions for weight loss [43] and found that interventions providing ‘accountability’ (i.e., the knowledge that someone else was looking at their weights) was associated with greater intervention effects [44]. Self-weighing with the addition of accountability may have been a factor in the control group’s low regain.

An alternative explanation for the lack of evidence for an effect on maintenance in the current trial may be that the low-intensity intervention involved insufficiently frequent participant contacts compared to previous, less scalable interventions. A meta-analysis has concluded that more intensive WLM interventions are not more effective than less intensive variants [4], but even the included ‘low intensity’ arms involved a greater number of in-person contacts than the present trial [13,19]. It is therefore possible that the intensity of the NULevel intervention was insufficient to affect regain. Although there is evidence for change in psychological outcomes, self-weighing, and physical activity, the effects might not have been strong enough to impact on the more distal measure of weight gain. Moreover, the greater levels of physical activity in the intervention group could have resulted increased energy consumption compared to the control group to compensate for excess energy expenditure.

The current trial is one of only four existing studies with long-term follow-up focused upon individuals who have lost weight entirely independently of the maintenance programme [11–13] and, as such, makes a valuable contribution to evidence on interventions for this group. Most individuals in the general population who attempt weight loss do so independently of professional or other support [45], yet previous maintenance interventions have overwhelmingly induced initial weight loss using standardised weight loss regimens. Such approaches result in study populations that do not accurately represent the many ways in which individuals lose weight [4]. By preferentially retaining individuals responsive to the initial weight loss programme they may also inflate maintenance effects because maintenance and weight loss intervention content frequently overlaps. The results of this study, therefore, may be more generalisable to the general population than those of the majority of previous trials. However, a limitation of the NULevel recruitment procedure is that participants were self-selected; participants reported having lost a significant amount of weight prior to trial entry and were highly motivated to continue managing their weight. This may be a reason for lower than expected weight regain in the current control group.

### Health economics

There was no evidence that the intervention was cost-effective. The within-trial analysis was conducted according to the rigorous and explicit standards expected of best practice methods [46]. No long-term modelling was conducted (as originally planned) because, based on the trial findings, it was considered not plausible that the extrapolation would change the conclusions of the within-trial analysis.

### Study strengths and limitations

Study strengths include the recruitment of a large community sample with independently achieved, objectively verified weight loss, a randomised design with sufficient power to detect clinically important weight difference, and a high rate of study retention over the 12-month period (92%). It was the first fully powered UK WLM trial with a 12-month follow-up. The NULevel intervention drew on the best available evidence, utilised self-regulatory behavioural strategies with proven effectiveness in WLM [11], and was developed in line with Medical Research Council guidelines for the development of complex interventions [16,20]. We used mobile internet technology, wirelessly connected weighing scales, and an interface providing automated, tailored SMS feedback, combined with a single face-to-face meeting, to achieve the delivery of an individualised, responsive intervention requiring a far smaller proportion of intervener time than in previous maintenance trials. Potential limitations are the use of connected study scales, which might have acted as an active intervention in the control arm and that all participants were recruited from the North East of England and may not generalise to other settings. Moreover, the study did not measure energy intake alongside physical activity as an outcome measure. Whilst we acknowledge that energy intake is the main determinant of obesity at the population level, the lack of available unobtrusive reliable measures of food intake, with sensitivity to change in trials, means that we cannot evaluate whether the NULevel intervention had an effect on energy intake.

### Conclusions

In conclusion, amongst individuals with obesity, we found no evidence of effectiveness of a remotely delivered, low-intensity behavioural intervention based on self-regulation theory in reducing weight regain compared to standardised lifestyle advice and independently achieved, clinically significant weight loss for individuals who received a set of wirelessly connected weighing scales. The NULevel intervention improved various hypothesised mediators compared to the control arm, including physical activity and self-weighing, but no differences in WLM. We conclude that the incremental dose of the NULevel intervention over the active control condition might have been insufficient to affect weight outcomes. This research should inform future intervention design decisions regarding delivery modality and intensity.

## Supporting information

S1 CONSORT Consolidated standards of reporting trials (CONSORT) checklist. (DOC)

S1 Appendix Links embedded in quarterly text messages sent to the control group. (DOCX)

S1 Protocol Study protocol. (PDF)

## Abbreviations

BCT: behaviour change technique
BMI: body mass index
BPM: beats per minute
CALO-RE: Coventry, Aberdeen & London-Refined
CONSORT: Consolidated Standards of Reporting Trials
ENRICHD: Enhancing Recovery in Coronary Heart Disease
GCSE/O-Level: General Certificate of Secondary Education/Ordinary Level
HND/A-Level: Higher National Diploma/Advanced Level
IMD: index of multiple deprivation
QALY: quality-adjusted life year
RCT: randomised controlled trial
SMS: short message service
SUR: seemingly unrelated regression
TAC/d: total activity counts per day
WLM: weight loss maintenance
