# Supplementary material for: Behavioural intervention for weight loss maintenance versus standard weight advice in adults with obesity: A randomised controlled trial in the UK (NULevel Trial)
Source: PLoS Med. 2019 May 7;16(5):e1002793. doi: 10.1371/journal.pmed.1002793 (PMC6504043; doi:10.1371/journal.pmed.1002793)
Supplement: S1 Appendix — (DOCX) [file pmed.1002793.s002.docx]

Appendices

1. Links embedded in quarterly text messages sent to the control group:
   1. <http://www.nhs.uk/Livewell/loseweight/Pages/Healthyfoodswaps.aspx>
   2. <http://www.nhs.uk/Livewell/loseweight/Pages/surprising-100-calorie-snacks.aspx>
   3. <http://www.nhs.uk/Livewell/Goodfood/Pages/food-labelling.aspx>
   4. <http://www.nhs.uk/Livewell/loseweight/Pages/Healthybreakfasts.aspx>
